# TRAIL and vitamins: opting for keys to castle of cancer proteome instead of open sesame

**DOI:** 10.1186/1475-2867-12-22

**Published:** 2012-06-06

**Authors:** Ammad Ahmad Farooqi, Shahzad Bhatti, Muhammad Ismail

**Affiliations:** 1 Lab for Translational Oncology and Personalized Medicine, Rashid Latif Medical College (RLMC), 35 km Ferozepur Road, Lahore, Pakistan; 2 Department of Human Genetics and Molecular Biology, University of Health Sciences, Lahore, Pakistan; 3 Institute of Biomedical and Genetic Engineering (IBGE), Islamabad, Pakistan

## Abstract

Cancer is a multifaceted molecular disorder that is modulated by a combination of genetic, metabolic and signal transduction aberrations, which severely impair the normal homeostasis of cell growth and death. Accumulating findings highlight the fact that different genetic alterations, such as mutations in tumor suppressor genes, might be related to distinct and differential sensitivity to targeted therapies. It is becoming increasingly apparent that a multipronged approach that addresses genetic milieu (alterations in upstream and/or parallel pathways) eventually determines the response of individual tumors to therapy. Cancerous cells often acquire the ability to evade death by attenuating cell death pathways that normally function to eliminate damaged and harmful cells. Therefore impaired cell death nanomachinery and withdrawal of death receptors from cell surface are some of major determinants for the development of chemotherapeutic resistance encountered during treatment. It is therefore essential to emphasize underlying factors which predispose cells to refractoriness against TRAIL mediated cell death pathway and the relevant regulatory components involved. We bring to limelight the strategies to re-sensitize TRAIL resistant cells via vitamins to induce apoptosis.

## Introduction

TRAIL plays an imperative role in host immunosurveillance in opposition to tumor progression, as it triggers apoptosis of tumor cells but not normal cells, and therefore has great therapeutic potential for cancer treatment. It is a well established fact that TRAIL binds to two cell-death-inducing (DR4 and DR5) and two decoy (DcR1, and DcR2) receptors. Various reviews have been written highlighting role of TRAIL mediated signaling cascade in prostate cancer [1-3]. Escalating preclinical studies reveal that the TRAIL ligand can efficiently induce cancer cell apoptosis. Completed and ongoing Phases I and II clinical trials using TRAIL, represent clinically promising outcomes exclusive of significant toxicity. To date, research has focused on the patterns of apoptosis induced by TRAIL and interpreting complex barcodes underlying TRAIL resistance. Escape of death receptors from cell surface is equivalent to narrow escape of cancerous cells. It is becoming progressively more obvious that membrane curvature is no longer viewed simplistically as a passive outcome of cellular activity. It has been appraised by considerable evidence as an active mode or alternatively a rheostat like switch to create membrane domains and to organize centres for membrane trafficking. The mechanism of internalization is instrumental in redistribution and reshuffling of versatile proteins in cytoplasm and plasma membrane in normal and cancerous cells. On a larger scale, membrane curvature occupies a central stage in growth and division however dysregulation in spatio-temporal pattern underlies cancer progression. This review untangles complex web of modulators which promote “duty shift” of the death receptors in the cancerous cells and the approaches which will counteract their withdrawal from the cell surface. In the next section we give a detailed account of Vitamins which are documented to stimulate the re-establishment of death receptors on plasma membrane and consequently re-sensitizing cancer cells to TRAIL mediated apoptosis.

### Vitamin A and TRAIL: two stepping stones of the TRAIL

Confluence of information suggests that underpinnings of refractoriness to TRAIL induced apoptosis in malignant cells and normal cells is not entirely understood. However substantial fraction of data has been added into the existing web of knowledge with reference to variety of factors that are present in the tumor microenvironment and can influence the response of malignant cells to TRAIL. An emerging paradigm in cancer chemoprevention is use of natural or biological substances to reverse, repress or counteract either the initial phase of carcinogenesis or the progression of neoplastic cells to cancer. This review provides a summary of the impact of vitamins on mechanisms connected with enhancement of TRAIL mediated signaling together with modulation of the apoptotic (death receptor expression, FLIP, and Bcl-2 or inhibitors of apoptosis (IAP) families) as well as cell signal transduction cascades.

It is becoming gradually more apparent that all trans retinoic acid (ATRA) restricted phosphorylation of FOXO3A in acute promyelocytic leukemia (APL) cells. The reduction in phosphorylation of FOXO3A resulted in its re-distribution by consequent shuttling into the nucleus to stimulate the expression of TRAIL [4]. Detailed investigations unveil the fact that refractoriness against TRAIL is generated by orchestrated activities of anti-apoptotic proteins. In accordance with this concept, recent information suggests that all-trans-retinyl acetate (RAc) trigger the expression of TRAIL death receptors and suppresses decoy receptors. Additionally, combinatorial approach induced apoptosis in APC-deficient premalignant cells without affecting normal cells in vitro Zhang et al., [5].

Promoter mapping, gel retardation and chromatin immunoprecipitation assays unraveled the detail that retinoids induced the expression of TRAIL mainly through crosstalk with NF-kappaB. However there is a differential mode of action in combination with etoposide, camptothecin or doxorubicin as it antagonized the apoptosis triggered by the chemotherapeutic drugs. Discordantly, apoptosis induced by TRAIL was not antagonized by retinoids [6].

Accumulating evidence suggests that that a combination treatment with all-trans retinoic acid (ATRA) and TGF-beta1 leads to remarkable ATRA-induced suppression of cell proliferation, which is simultaneously accompanied by repression of ATRA-induced apoptosis in human leukemia HL-60 cells. Henceforth TGF signal transduction cascade resulted in dampening apoptosis inducing activity of ATRA by enhancing expression of c-FLIP(L) protein [7].

It is worth mentioning that retinoic acid (RA) has antileukemic activity and up-regulates the expression of TRAIL and its DR4 and DR5 receptors. In a RA-sensitive cell line PLB985, targeted inhibition of TRAIL blocked RA-induced apoptosis [8].

It is intriguing to note that in the leukemic blasts of acute promyelocytic leukemia patients, retinoic-acid-induced expression of TRAIL caused blast apoptosis. Therefore, initiation of TRAIL-mediated death signaling is contributory to the therapeutic value of retinoids [9,10]. Comprehensive analysis dismantled the fact that many mechanisms accounted for the anticarcinogenic actions of dietary constituents. Future studies must converge on multiple intracellular-signal transduction cascades as common molecular targets for vitamin A.

### Vitamin C and TRAIL: it takes two to tango

The antioxidant, vitamin C stimulated perifosine (an alkylphospholipid tested in phase II clinical trials)-induced expression of DR4 and DR5 in head and neck squamous cell carcinoma cells and inhibited the growth of xenografts [11]. Controversies exist with reference to role of Vitamin C in induction of apoptosis as it impedes the elevation of reactive oxygen species (ROS) levels induced by TRAIL and impairs caspase-8 activation [12]. Consistent with the same interpretation, Vit C abrogated triterpenoid methyl-2-cyano-3, 12-dioxooleana-1, 9-dien-28-oate (CDDO-Me) induced apoptosis [13]. In vitro analysis still is insufficient to provide a detailed concept of effect of vitamin C on TRAIL mediated signaling cascade and its respective receptors. Moreover, keeping in view, divergent effects of vitamin C, it will have differential effects on individuals whose genotype enhances significant benefit derived from an increased intake of vitamin C enriched items, and a small segment of the population that may be disadvantaged. Therefore, large-scale, whole-genome association studies will surely present a reasonable understanding of the steps and factors that modulate machinery of apoptosis.

### Vitamin D

Considerable verification suggests that pretreatment with 1,25-dihydroxyvitamin D3 repressed apoptosis induced by TRAIL and Fas ligand by up-regulating decoy receptor and death receptor 4 and repressing Fas. However abrogation of death receptor 4 or enforced expression of Fas attenuated the suppressive effect of 1,25-dihydroxyvitamin [14]. On a parallel note, PEA-15 (phosphoprotein enriched in astrocytes-15 kD, also known as PED), has anti-proliferative and anti-apoptotic effects and is transcriptionally triggered by vitamin D(3). Outstandingly, vitamin D(3) was able to chaperone cells from TRAIL-induced apoptosis by stimulating the expression of the PEA-15 [15].

It has recently been explored that TRAIL mediated apoptosis is impaired by IL-1beta release from macrophages. Contrarily, Vitamin D(3) interfered with the release of IL-1beta from macrophages thus re-sensitizing tumor cells to TRAIL-induced apoptosis [16]. Center of attention in future work should be on identifying 'driver' molecular defects of oncogenic pathways that can be targeted therapeutically, discovering predictive biomarkers for treatment response, and prioritizing promising drugs to speed up their approval.

An enhanced appreciation of the molecular biology of cancer cell growth and survival and the role of the microenvironment in supporting the survival of cancer cells in future will result in identification of additional distinct roles of vitamin D in regulating cellular activity.

### Vitamin E: travel guide or pathfinder

It is perceptible that α-TOS and hrTRAIL in combination act with remarkable synergy to induce apoptosis in breast cancer cells. Moreover, α-TOS repressed the expression of FLIP and c-IAP1 in erbB2-positive cells [17].

In agreement with the same conception, Vitamin E derivative RRR-α-tocopherol ether-linked acetic acid analog (α-TEA) stimulated the expression of TRAIL and DR5and considerably repressed levels of antiapoptotic factor, c-FLIP L [18,19]. Further insights into the mechanisms indicated that alpha-TEA induced suppression of c-FLIP (L) protein level was triggered by JNK/CHOP/DR5 loop via a JNK dependent Itch E3 ligase ubiquitination that promoted JNK/CHOP/DR5 amplification loop by restricting c-FLIP's inhibition of caspase-8 [20].

Another interesting finding suggests that Tocotrienol (T3), an unsaturated vitamin E but not tocopherol, induced transcriptional up-regulation of the death receptor (DR)-4 and DR5 via enhanced production of reactive oxygen species and augmented activation of ERK1 [21]. Analogously, treatment of human MDA-MB-231 and MCF-7 cells with γ-T3 resulted in activation of JNK and p38 MAPK, and upregulated DR5 and C/EBP homologous protein (CHOP). Insightful approaches suggested that DR5 was transcriptionally regulated by CHOP after γ-T3 treatment [22].

It is becoming progressively more appreciable that alpha-tocopheryl succinate (alpha-TOS) re-sensitizes TRAIL-resistant malignant mesothelioma (MM) cells to TRAIL-induced apoptosis. This transformation of TRAIL refractory (MM) cells to TRAIL sensitive (MM) cells occurs in a p53-dependent manner as p53competent MM cells are responsive to sensitization however, p53deficient counterparts are insensitive [23]. Furthermore, malignant(MM) and non-malignant counterparts (Met-5A) have differential responsiveness to alpha-tocopheryl succinate (alpha-TOS) and TRAIL. Molecular approaches suggest that MM cells are susceptible to alpha-TOS and less to TRAIL, whereas Met-5A cells are susceptible to TRAIL and resistant to alpha-TOS [24] . High throughput technologies indicated that alpha-TOS and TRAIL acted synergism to kill MM cells via mitochondrial pathway, and were nontoxic to nonmalignant mesothelial cells [25]. It is attention-grabbing that mitochondrion is instrumental in apoptosis induced by alpha-tocopheryl succinate as mtDNA-deficient cells display resistance to alpha-TOS [26].

Mounting data suggests that tocotrienol-induced caspase-8 activation and apoptosis in malignant (+)SA mammary epithelial cells is irrespective of the activation of death receptors and is dependent on suppression of the PI3K/PDK/Akt signal transduction cascade and succeeding repression in FLIP expression [27].

It is becoming more and more evident that exposure of the cells to TRAIL results in transient activation of NF-kappaB, a process that is suppressed by cell pretreatment with alpha-TOS. alpha-TOS re-sensitizes cells to TRAIL killing, through repression of NF-kappaB activation [28].

It is worth mentioning that alpha-TOS is nontoxic to normal cells however simultaneously triggers apoptosis in p53 deficient and p21deficient cancer cells [29].

It is contemplating to note that multiple gene variants that have small to moderate individual phenotypic effects contribute to the overall progression of cancer. A particular individual's level of cancer susceptibility and recurrence depends on interactions between environmental factors and a wide range of modifier genes, which systematically impact derailed cellular activities and resistance against a multiple therapeutic interventions especially TRAIL. The latest genome-wide association studies in large cohorts of patients with different cancers provided new insights into the pathophysiology of these molecular disorders and have suggested contribution of previously unsuspected molecular signaling pathways. Additionally, model studies have identified supplementary susceptibility genes for inducing resistance against TRAIL mediated apoptosis. We therefore developed a roadmap of various signaling pathways which were contributory in driving or suppressing carcinogenesis alongwith effect of vitamins at nanoscale level. Next, we discuss the factors governing sequestration of death receptors off plasma membrane.

### Death receptors internalization: gatekeepers are hijacked

**Figure 1:**
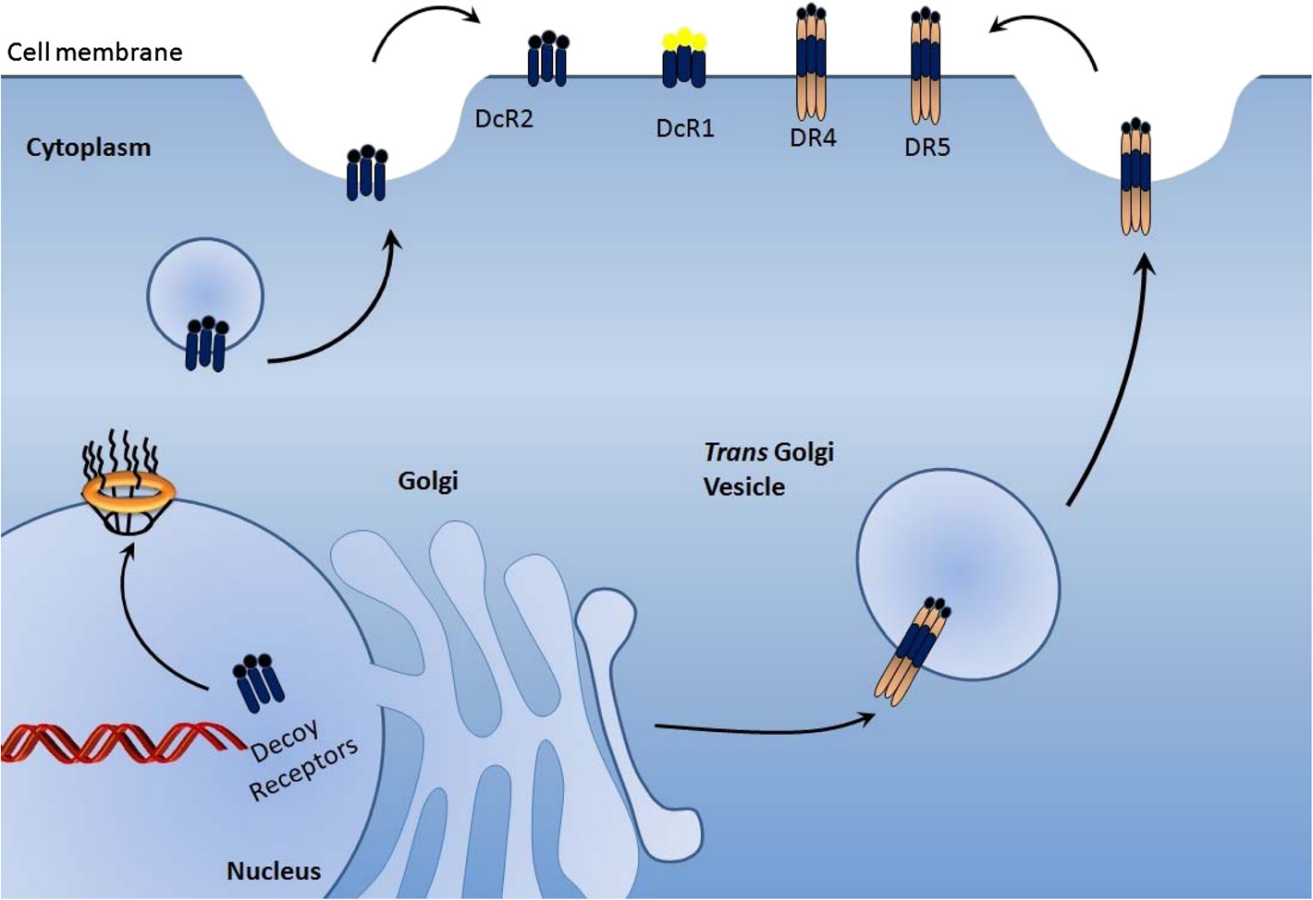
Modes of internalization of death receptors.

It is praiseworthy that new techniques are deepening our insight into the dynamics of membrane organization. Here, we discuss how the field of internalization and degradation of death receptors has matured and present growing model in which membranes are occupied by fluctuating nanoscale assemblies of sphingolipids, cholesterol and proteins that can be stabilized into platforms that are important in TRAIL mediated signaling and membrane trafficking. TRAIL mediated signaling occurs by various mechanisms, which can be divided into those that are clathrin dependent and those that are clathrin independent Figure 1.

Emerging evidence suggests that stable TRAIL-resistant sub-population of the metastatic colon cancer cell line LIM6-TR displays an increased cell-surface expression of galectin-3. Interestingly, protein purification strategies indicated that galectin-3, an endogenous lectin, co-precipitated with death receptors. Targeted inhibition of galectin-3 re-sensitized resistant cells to TRAIL and facilitated TRAIL-mediated endocytosis of TRAIL/death receptors complexes [30]. It is getting gradually more obvious that ARAP1 knockdown drastically hampers cell surface expression of DR4 in several tumor cell lines and represses their TRAIL-induced death. Laboratory investigations suggested that transfected ARAP1 co-purified with DR4 and co-occured with it in the endoplasmic reticulum/Golgi, at the cytoplasmic membrane and in early endosomes of TRAIL-treated cells [31].

Additionally, TRAIL cytotoxicity in hepatocellular carcinoma cells is triggered by lysosomal permeabilization. Death receptors behave in a differential manner in terms of trafficking to lysosomes as DR5 complex under go immediate endocytosis upon ligand stimulation, while DR4 complex is not proficiently internalized. Upon TRAIL treatment, DR5 co-existed with lysosomes following internalization and abrogation of DR5 trafficking to lysosomes in Rab7 deficient cells also repressed TRAIL-mediated lysosomal disruption and apoptosis [32]. Emerging line of evidence indicated that PRMT5 selectively interacted with death receptor 4 and death receptor 5. Gene silencing strategies suggested that PRMT5 abrogation sensitized various cancer cells to TRAIL. PRMT5 effectively modulated TRAIL-induced activation of inhibitor of kappaB kinase (IKK) and nuclear factor-kappaB (NF-kappaB), thus stimulating the expression of several NF-kappaB target genes [33].

Moreover, in MDA-MB-231 breast cancer cells, endocytosed DR4 is cleaved in a caspase-dependent manner. It is worth mentioning that receptor internalization does not inhibit caspase activation, instead stimulates apoptosis after TRAIL treatment. Consistent with the same concept, it is obvious that TRAIL-induced receptor endocytosis suppresses its pro-apoptotic potential [34].

Besides, impairment of endocytosis or disruption of clathrin-dependent endocytosis signaling components (adaptor protein 2 and clathrin) recapitulates cell surface appearance of the death receptors and sensitize TRAIL-resistant cells to TRAIL-induced apoptosis. Structural overview of DR4 indicates that endocytosis is modulated by its cytoplasmic domain EAQC(337)LL. Therefore unquestionably clathrin-mediated endocytosis pathway could be a prospective target for therapeutics to circumvent tumor resistance to TRAIL receptor-targeted therapies [35].

It is becoming sequentially more apparent that death receptors trigger selective destruction of the clathrin-dependent endocytosis nano- machinery. DR stimulation initiated instantaneous, caspase-mediated cleavage of clathrin-pathway apparatus, impeding retrieval of cargo protein transferrin. Analogously, caspases cleaved the clathrin adaptor subunit AP2alpha between characteristically discrete domains, while effector caspases processed clathrin's heavy chain [36].

Escalating evidence suggests that COX-2 abrogation sensitizes human colon carcinoma cells to TRAIL induced apoptosis by enhancing assembly of the TRAIL receptor DR5 at the cell surface. Simultaneously, it is also associated with reorganization of the death-inducing signaling complex components into cholesterol-rich and ceramide-rich domains known as caveolae. This progression is dependent on accumulation of arachidonic acid and consequent activation of acid sphingomyelinase for the generation of ceramide within the plasma membrane outer leaflet [37].

It is intriguing to note that enforced expression of caveolin results in de-sensitization of cells towards TRAIL as engagement of TRAIL with its respective receptor DR4 reduces the localization of DR4 in caveolae and results in its internalization. Obstruction of caveolae-mediated internalization of DR4 by filipin III outstandingly improved TRAIL-induced apoptosis [38].

Post translational modifications of proteins underpin membrane-protein association and influence protein trafficking, stability or aggregation, thus playing an important role in protein signalling. In accordance with this assumption recent data suggests that DR4 is palmitoylated, whereas DR5 and TNFR1 are not. In addition, DR4 palmitoylation is essential for its raft localization and its ability to undergo oligomerization [39].

Consistently, Ursodeoxycholic acid (UDCA) triggered formation of lipid rafts via ROS production/PKCδ activation pathway that played fundamental roles in UDCA-induced apoptosis. Lipid rafts are essential not only for provision of a platform for DR5 action but also for regulation of DR5 expression [10]. Taken together, epirubicin also significantly augmented lipid raft DR4 and DR5 aggregation, alongwith the localization of DR4 and DR5 in the lipid rafts [40].

While drawing a parallel between adherent and detachment cells apoptosis assay revealed that detached cells are more sensitive to DR5 antibody-induced apoptosis than adherent cells. As transition from adhesion to detachment of EC9706 cells results in DR5 relocalization, and enhances cytoplasmic translocation of DR5 to cell surfaces via a Golgi-dependent pathway [41].

Cellular organelles in the endocytic pathways have a characteristic spatial distribution and communicate through a highly structured system of vesiculo-tubular transport. TRAPP proteins harmonize consecutive stages of transport, such as vesicle formation, vesicle motility and tethering of vesicles to their target compartment. These protein complexes are well compartmentalized in organelle membranes, making them exceptional mediators for determining transport specificity.

**Figure 2:**
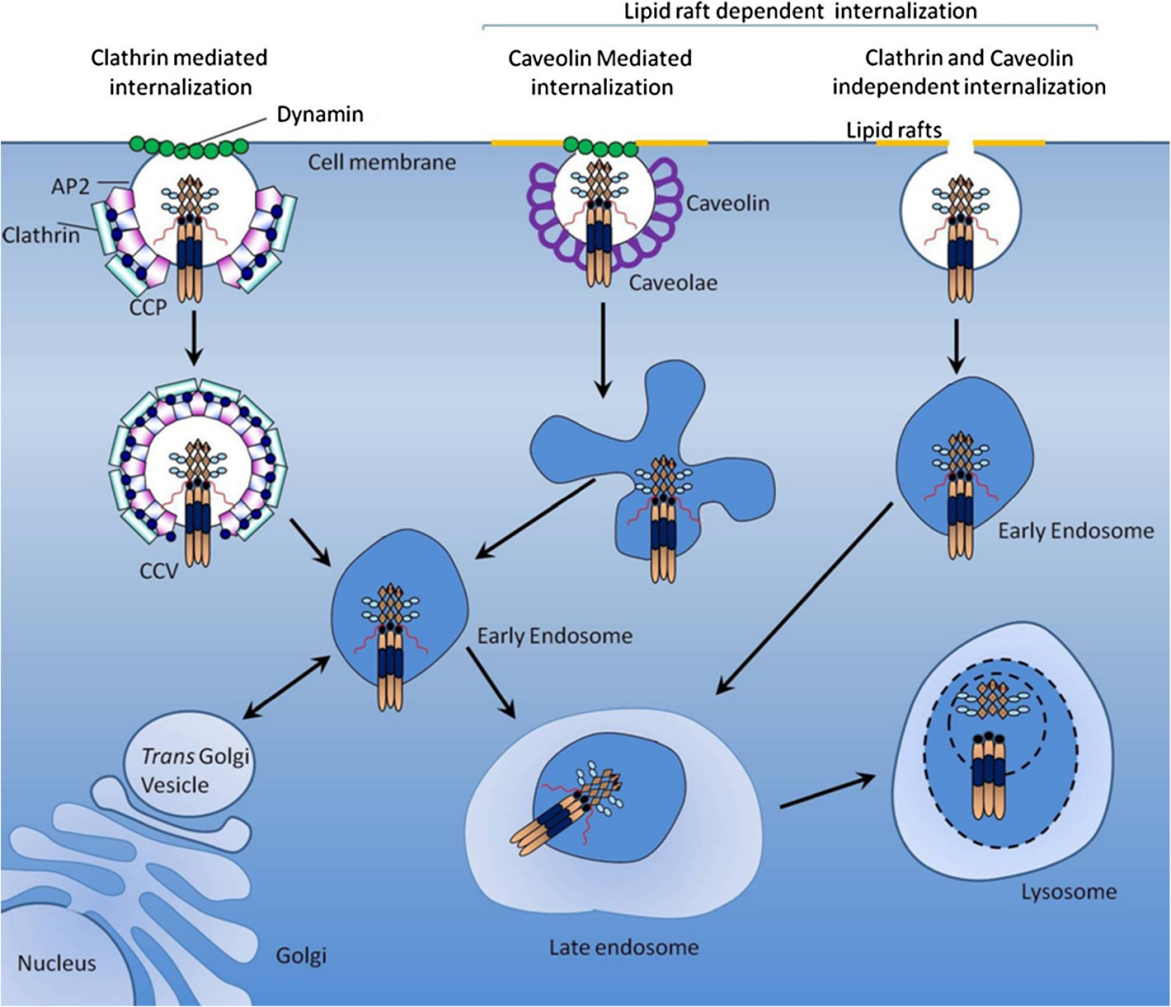
Various routes of translocation of death receptors and decoy receptors towards cell surface.

A study delineating localization patterns of the receptors in melanoma cells by confocal microscopy suggested that TRAIL-R1 and R2 were located in the trans-Golgi network, but the inhibitory receptors TRAIL-R3 and -R4 were located in the nucleus Figure 2. Signals from TRAIL-R1 and -R2 tactfully triggered redistribution of TRAIL-R3 and -R4. It was suggested by the studies that cells that represented only TRAIL-R3 receptors and not TRAIL-R1 or -R2 did not undergo repositioning from the nucleus and was verified by use of mAbs which blocked interaction of TRAIL with TRAIL-R1 and -R2. On the contrary, in one cell line which expressed only TRAIL-R2 and -R3 the redistribution of R3 was completely inhibited by mAb against TRAIL-R2. In the similar manner, in a line (Me4405) which expressed all TRAIL-R, inhibition of TRAIL-R2 alone was insufficient to stop relocation of TRAIL-R3 and -R4, and it was also essential to inhibit TRAIL-R1. These results documented that the signals for relocation of the decoy receptors are dependent on signal transduction cascades from TRAIL-R1 or -R2. [42]. Additionally, there are some other regulators which mediate trafficking of death receptors to cell surface. It has presently been recognized that golgi-specific Asp-His-His-Cys (DHHC) zinc finger protein (GODZ) regulates TRAIL/DR4-mediated apoptosis. Studies indicated that GODZ made its attachment to DR4, but not to DR5, through the DHHC and the C-terminal transmembrane domain. Moreover, GODZ triggered localization of DR4 to the plasma membrane (PM) via DHHC motif as proof of concept was provided by mutation studies which confirmed that introduction of mutation into the cysteine-rich motif of DR4 resulted in its mistargeting and attenuated TRAIL- or GODZ-mediated apoptosis [43].

The signal recognition particle complex (SRP) is a molecular machinery of the “signal hypothesis” that is contributory in protein sorting through the secretory pathway. Similarly, experiments demonstrated that expression of the signal recognition particle (SRP) complex was vital for apoptosis mediated by DR4, but not DR5. Targeted inhibition of SRP subunits by RNA interference resulted in a remarkable decrease in cell surface DR4 receptors that interrelated with inhibition of DR4-dependent cell death. Expression of cell surface DR4 was severely repressed in cells lacking expression of SRP54 or SRP72 [44]. Membrane proteins are embedded into the endoplasmic reticulum (ER) by highly precise and orchestrated pathway. It is imperative to detect that co-translational pathway uses signal recognition particle (SRP) and its receptor for targeting and the SEC61 translocon for membrane integration. It is appealing to consider that translational pathway uses an entirely different set of factors involving transmembrane domain (TMD)-selective cytosolic chaperones and an accompanying receptor at the ER with reference to death receptor. Clarification of the structural and mechanistic beginning and intricacy of this post-translational death receptor protein insertion pathway will doubtlessly highlight some more unexplained facts.

It is imperative to explore whether DR4 is indeed translocated to the Golgi and the impairment of migratory pathway to properly traffic to the plasma membrane is a secondary effect of SRP depletion. Likewise, it will be essential to find out how DR4 and other proteins are translocated to the Golgi and whether these events are dependent on chaperones or the ER.

Keeping in view the trafficking of receptors from endoplasmic reticulum to golgi and later to plasma membrane is a well organized mechanism. We have several hints of protein complexes which might play a role in the translocation. TRAPP proteins are identified to be component of a large multi-protein complex involved in ER-to-Golgi and intra-Golgi trafficking. Details were comprehensively reviewed by [45]. For instance, TRAPPCI is necessary for the recruitment of ER-derived vesicles to the cis-Golgi. Another protein, TRAPPC4 may play a role in nucleocytoplasmic transport in colorectal cancer as enforced expression of TRAPPC4 correspondingly enhanced activation ERK1/2 and its consequent movement into the nucleus [46].

Recent molecular genetics study indicated that TRAPPC9 encoded the NIK- and IKK-beta-binding protein (NIBP) [47]. NIBP interacted with NIK, IKK(beta), but not IKK(alpha) or IKK(gamma). Moreover enforced expression of NIBP potentiated tumor necrosis factor-alpha-induced NF-kappaB activation through augmented phosphorylation of the IKK complex and its downstream I(kappa)B(alpha) and p65 substrates. On the contrary, impairment of NIBP expression reduced tumor necrosis factor-alpha-induced NF-kappaB activation and decreased Bcl-xL gene expression in cancer cells [48].

It is indubitable that despite tremendous information added into the developing landscape of death receptor trafficking pathways many protein complexes and assemblies are still lost and a better concept of multi-component, multi-layered trafficking pathway is required. How TRAPP proteins interact with death receptors and facilitate or repress migration from endoplasmic reticulum to golgi and further towards plasma membrane is a missing link. As a consequence, supplementary structure-function studies will be needed to describe the particular sequences necessary for DR4 cell surface localization. In this regard, how DR5 and other TNF family receptors find their way to the plasma membrane remains fascinating, yet an outstanding and under-defined question.

Accumulating evidence using confocal microscopy suggested that DR5 were localized in the nucleus in HeLa and HepG2 cells. Nuclear trafficking of DR5 was inhibited in the cells deficient for importin β1 however enforced expression of importin β1 restored redistribution of DR5 in nucleus [49]

It is imperative to note that certain viral proteins counteract killing through death receptors by maximizing internalization and degradation. In agreement with this interpretation, human adenovirus type 5 encodes three proteins, named RID suppressed TRAIL-induced apoptosis of infected human cells. RID promoted endocytosis of TRAIL-R1 from the cell surface, as indicated by flow cytometry and indirect immunofluorescence studies for TRAIL-R1. TRAIL-R1 was internalized in discrete vesicles and degradation of TRAIL-R1 was noted [50].

Correspondingly, localization of DR4 but not DR5 in lipid rafts was mediated by TRAIL and fludarabine treatment [51].

It is also necessary to make a note of Nitrosylcobalamin (NO-Cb) that inhibits survival signaling by enhancing drug efficacy by preventing concomitant activation of negative regulators of apoptosis (NF-kappaB or AKT). Additionally, use of NO-Cbl and Apo2L/TRAIL represents a promising anti-cancer combinatorial drug because DR4 (TRAIL R1) is S nitrosylated following NO-Cbl treatment. S-nitrosylation at cysteine 304 facilitates relocalization of Fas to lipid rafts, formation of the death-inducing signalosome, and induction of cell death[52-55].

## Conclusions

It is undeniable that nutrigenomics has emerged as an important facet of molecular biology and employs high-throughput genomics technologies to disentangle how nutrients modulate gene and protein expression and eventually influence cellular and organism metabolism. It is vital to use and integrate nutrigenomics in all future nutrition studies portray broader landscape for evidence-based nutrition in near future. Concordant with same viewpoint, whole-genome sequencing is possible, comprehensive strategies for integrating genomic data and counseling of patients need to be developed.

Incontrovertibly, vitamins exert pleiotrophic responses in malignant cells leading to cell cycle arrest, differentiation, and apoptosis. Apart from their implications in killing cancer cells via apoptosis, vitamins regulate expression of genes involved in cell proliferation and cell death in a "subapoptotic" manner. For instance, these vitamins mediate the cell cycle machinery, resulting in cell cycle arrest. We have elucidated an overview of cooperative antitumor effect when vitamins are combined with immunological agents. Vitamins and TRAIL synergize to kill cancer cells either by stimulating the expression or appearance of death receptors on cell surface respectively or by amplifying the mitochondrial apoptotic pathway without being toxic to normal cells. Lipophilic vitamins are being explored for their anticancer properties by many researchers [56]. Silica nanoparticles are also a worthwhile addition in medicine [57]. Moreover it is important to outline those proteins which negatively modulate anti-apoptotic proteins like TRIM32 which degrades X-linked inhibitor of apoptosis (XIAP) and sensitizes cells to tumor necrosis factor (TNFα)-induced apoptosis.

It is significant that a single-agent 'magic' pill for chemoprevention is not highly effective and that using different combinations of phytochemicals and vitamins may be the answer.

## Competing interests

The authors declare that they have no competing interests.

## Authors’ contributions

All authors contributed equally to the development of the ideas and the writing of this review. All authors read and approved the final manuscript.
